# Deciphering the mode of action and position of genetic variants impacting on egg number in broiler breeders

**DOI:** 10.1186/s12864-020-06915-1

**Published:** 2020-07-24

**Authors:** Eirini Tarsani, Andreas Kranis, Gerasimos Maniatis, Santiago Avendano, Ariadne L. Hager-Theodorides, Antonios Kominakis

**Affiliations:** 1 Department of Animal Science, Agricultural University of Athens, Iera Odos 75, 11855 Athens, Greece; 2 Aviagen Ltd., Newbridge, Midlothian, EH28 8SZ UK; 3 The Roslin Institute, University of Edinburgh, Midlothian, EH25 9RG UK

**Keywords:** Egg number, Broilers, Additive and dominant effects, Prioritization analysis, Genome-wide association study

## Abstract

**Background:**

Aim of the present study was first to identify genetic variants associated with egg number (EN) in female broilers, second to describe the mode of their gene action (additive and/or dominant) and third to provide a list with implicated candidate genes for the trait. A number of 2586 female broilers genotyped with the high density (~ 600 k) SNP array and with records on EN (mean = 132.4 eggs, SD = 29.8 eggs) were used. Data were analyzed with application of additive and dominant multi-locus mixed models.

**Results:**

A number of 7 additive, 4 dominant and 6 additive plus dominant marker-trait significant associations were detected. A total number of 57 positional candidate genes were detected within 50 kb downstream and upstream flanking regions of the 17 significant markers. Functional enrichment analysis pinpointed two genes (*BHLHE40* and *CRTC1*) to be involved in the ‘entrainment of circadian clock by photoperiod’ biological process. Gene prioritization analysis of the positional candidate genes identified 10 top ranked genes (*GDF15, BHLHE40, JUND, GDF3, COMP, ITPR1, ELF3, ELL, CRLF1* and *IFI30).* Seven prioritized genes (*GDF15, BHLHE40, JUND, GDF3, COMP, ELF3, CRTC1)* have documented functional relevance to reproduction, while two more prioritized genes (*ITPR1* and *ELL*) are reported to be related to egg quality in chickens.

**Conclusions:**

Present results have shown that detailed exploration of phenotype-marker associations can disclose the mode of action of genetic variants and help in identifying causative genes associated with reproductive traits in the species.

## Background

The breeding objective used for selection in broilers is balanced between reproduction, welfare and production traits [1]. Modern broiler breeding programs strive to optimize the overall reproductive efficiency, which is defined as the number of viable chicks per breeder hen and is determined by the egg production in combination with fertility and hatchability. Among the different metrics to describe egg production, egg number (EN), defined as the number of eggs laid over the duration of the laying period (from 28 to 54 weeks), is one of the most commonly used ones for selection purposes in commercial broilers [2, 3].

As a typical reproductive trait, EN presents low to medium additive heritability estimates. In broiler hens, pedigree-based additive heritability for the trait has been estimated as high as 0.32, while respective estimates are in the range from 0.13 to 0.36 when using genomic relationship matrices [3, 4]. The contribution of dominance may also be of importance for the trait, as estimates of the genomic dominant heritability has been found as high as 0.06 [3].

High-density SNP (single nucleotide polymorphism) genotyping arrays have greatly facilitated the detection of candidate causal variants in genome-wide association studies (GWAS) for various traits related to egg production and egg quality. Most GWAS have, so far, focused on the detection of additive SNPs for egg production [5–7] and egg quality traits [5, 7–10]. It is noted that these studies have been focusing on EN in layer chickens and not broiler breeders. Moreover, to our knowledge, there is only one study [7] that aimed at identifying dominant SNPs for egg production and quality traits in chickens.

Driven from the scarcity of published reports for broiler breeders, we elaborated the present study with the primary aim to detect genetic variants impacting on EN. Next, we sought to describe the mode of gene action of the significant genetic variants and finally attempted to provide a list with most likely candidate genes for the trait under investigation. Current findings are expected to contribute to a better understanding of the genetic mechanism(s) underlying the EN phenotype in the species.

## Results

### Significant SNPs and PVE

**Fig. 1:**
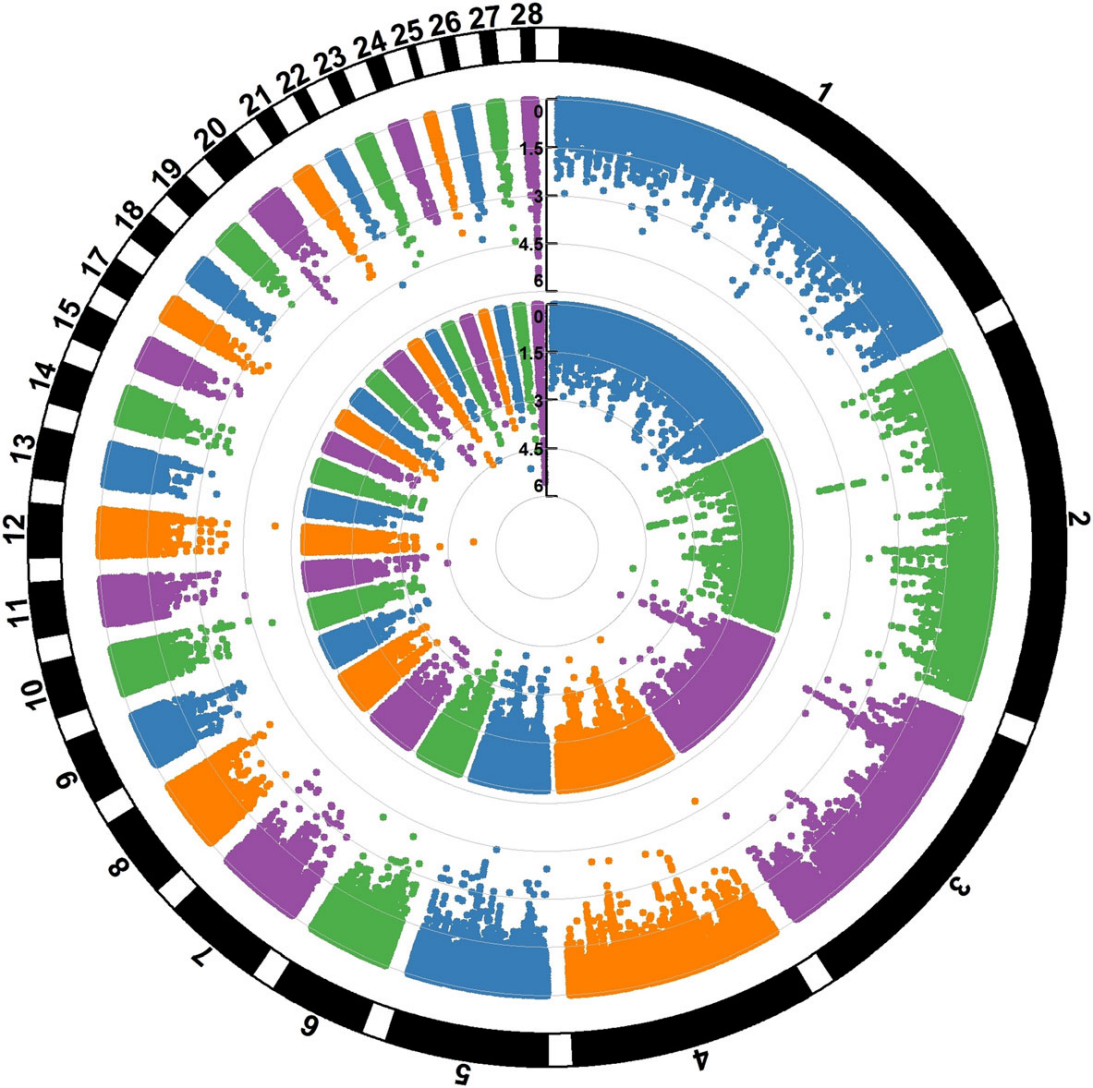
Circular Manhattan plot displaying the chromosome-wide significant associations for EN. The −log_10_(*p*-values) of the additive (inner circle) and dominant (outer circle) SNPs are shown across the 28 autosomal chromosomes. This plot was constructed with the CMplot package (https://github.com/YinLiLin/R-CMplot) in R (http://www.r-project.org/)

**Fig. 2:**
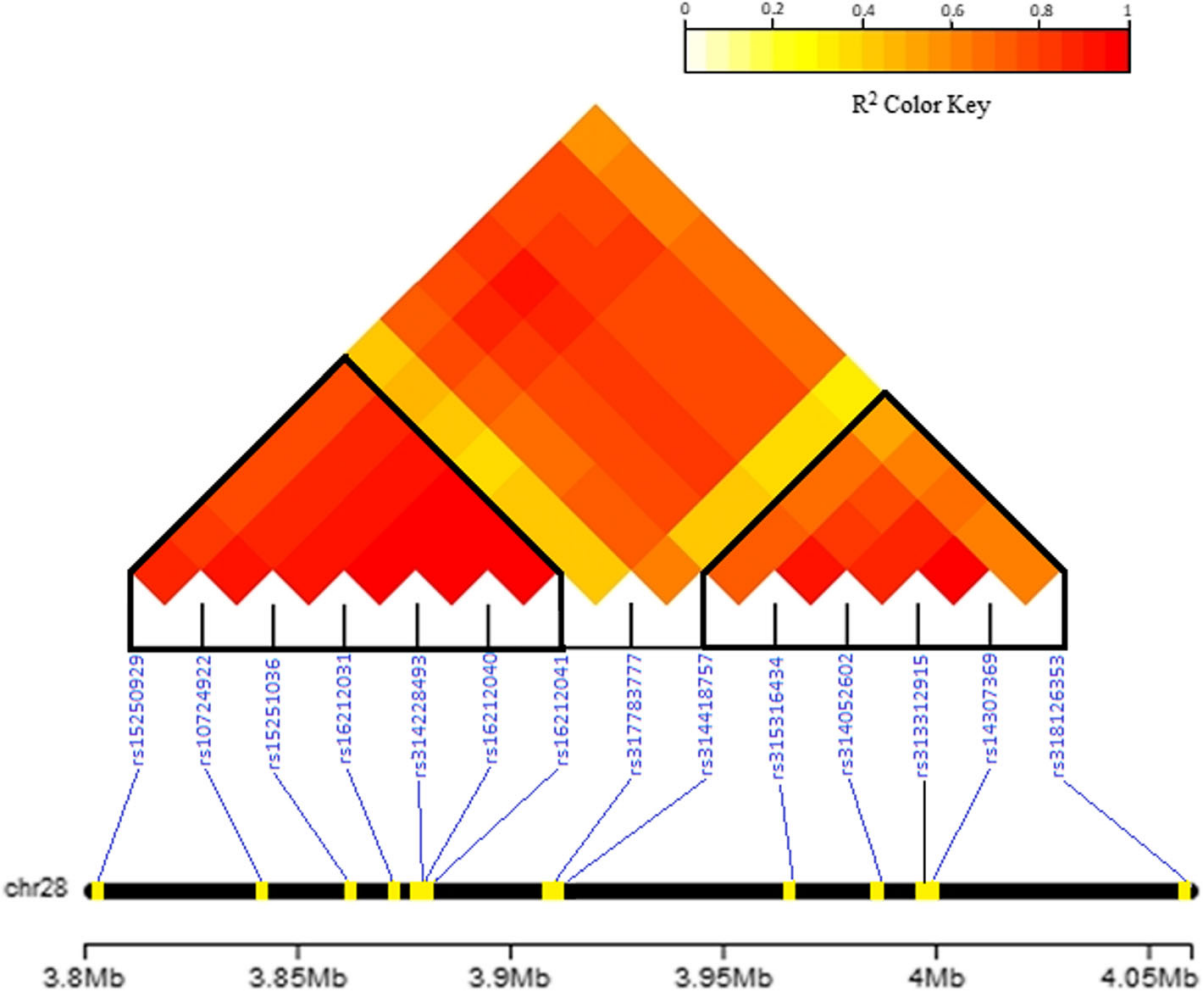
LD heatmap for the 14 SNPs (blue labels) on GGA28. Note the formation of 2 LD blocks (denoted as black lined polygons). LD levels were estimated using the gaston R package and were graphically displayed with use of LDheatmap [11] package in R (http://www.r-project.org/)

Additive and dominant genomic heritability estimates were identical and equal to 0.167 (SE = 0.03) for the trait. The Q-Q plots (see Supplementary Fig. 1, Additional file 1) of the expected and observed SNP *p*-values along with estimates of the genomic inflation factors (*λ* = 0.95 and 0.97 for the respective additive and dominant genetic model) were indicative of no systematic bias due to population structure or analytical approach. Profiles of the SNP *p*-values (expressed as -log_10_) for the additive and dominant genetic model are presented in form of circular Manhattan plots in Fig. 1. No SNP was found to reach genome-wide significance (*p* < 2.09E-07) using the Bonferroni correction method. Nevertheless, using the same correction method, a total number of 17 SNPs reached chromosome-wide significance across four autosomes (12, 22, 26 and 28) (Table 1). Specifically, one marker (*rs313298834)* was detected on GGA12 (threshold *p* = 0.05/7475 = 6.68896E-06), one (*rs314011910)* on GGA22 (threshold *p* = 0.05/1870 = 2.6738E-05), one (*rs313045367)* on GGA26 (threshold *p* = 0.05/3013 = 1.65948E-05) and 14 on GGA28 (threshold *p* = 0.05/2268 = 2.20459E-05). As observed in Table 1, 7 SNPs were associated with additive, 4 SNPs with dominant and 6 markers with both gene actions. Of the additive SNPs, one marker (*rs313045367*) resided on GGA26 while 6 were located on GGA28. One dominant SNP (*rs314011910)* was detected on GGA22 while 3 dominant SNPs (*rs15250929, rs314052602* and *rs318126353*) were located on GGA28. Of markers displaying both gene actions, one marker (*rs313298834*) resided on GGA12 and 5 were located on GGA28 (Table 1). Note that the 14 significant SNPs residing on GGA28 were co-localized in a region spanning 240,432 bp (3,818,934-4,059,366 bp) with high LD (r^2^) levels. A detailed view of these SNPs along with LD (r^2^) levels between markers is depicted in Fig. 2. As the LD heatmap shows, there are two haplotype blocks (r^2^ > 0.70) formed by marker pairs *rs15250929-rs16212041* and *rs314418757-rs318126353* (Fig. 2). PVE by significant markers ranged from 0.70% (*rs10724922, rs317783777*) to 0.85% (*rs314418757*) for the additive markers and from 0.69% (*rs314011910*) to 0.84% (*rs16212031, rs16212040, rs16212041*) for the dominant markers (Table 1). All together, the significant additive and dominant SNPs explained a considerable part i.e. 60 and 47% of the additive and dominant genomic heritability, respectively. Nevertheless, as many of the significant markers were localized in nearby locations on GGA28, PVE by markers are biased upwards.

**Table 1:** Chromosome-wide significant SNPs identified by additive (add), dominant (dom) or both additive and dominant (add/dom) genetic models (MAF: Minor Allele Frequency)

SNP ID	GGA^**a**^	Position (bp)^**b**^	***p***-value (add/dom)	-log_**10**_(***p***-value) (add/dom)	Minor allele	MAF	PVE^**c**^(%)		Genetic model
							add	dom	
*rs313298834*	12	18,995,645	5.03832E-06/3.37289E-06	5.298/5.472	B	0.34	0.8	0.83	add/dom
*rs314011910*	22	1,711,605	2.30566E-05	4.637	A	0.14	–	0.69	dom
*rs313045367*	26	362,590	8.41209E-06	5.075	B	0.15	0.77	–	add
*rs15250929*	28	3,818,934	1.93573E-05	4.713	B	0.2	–	0.7	dom
*rs10724922*	28	3,855,714	2.0557E-05	4.687	A	0.21	0.7	–	add
*rs15251036*	28	3,875,127	1.74651E-05	4.758	B	0.21	0.71	–	add
*rs16212031*	28	3,885,458	5.56121E-06/3.19578E-06	5.255/5.495	A	0.2	0.80	0.84	add/dom
*rs314228493*	28	3,888,943	4.69378E-06/4.46165E-06	5.328/5.351	B	0.2	0.81	0.81	add/dom
*rs16212040*	28	3,892,786	3.35519E-06/3.06323E-06	5.474/5.514	B	0.2	0.83	0.84	add/dom
*rs16212041*	28	3,892,872	3.24649E-06/3.06323E-06	5.489/5.514	A	0.2	0.83	0.84	add/dom
*rs317783777*	28	3,919,505	2.02328E-05	4.694	A	0.14	0.70	–	add
*rs314418757*	28	3,921,905	2.70727E-06/1.62505E-05	5.567/4.789	A	0.21	0.85	0.72	add/dom
*rs315316434*	28	3,971,928	1.71745E-05	4.765	A	0.22	0.71	–	add
*rs314052602*	28	3,990,564	1.30589E-05	4.884	B	0.21	–	0.73	dom
*rs313312915*	28	3,999,772	8.56382E-06	5.067	B	0.22	0.76	–	add
*rs14307369*	28	4,003,865	1.37243E-05	4.863	A	0.21	0.73	–	add
*rs318126353*	28	4,059,366	5.35831E-06	5.271	B	0.23	–	0.80	dom

^a^ Chromosome for *Gallus gallus*

^b^ Positions were based on *GRCg6a* assembly

^c^ Proportion of variance explained

### Estimation of degree of dominance

Application of the LASSO method on the 14 co-localized SNPs on GGA28 resulted in selection of two markers i.e. *rs16212040* and *rs318126353* each one residing per different LD block (Fig. 2). Of these, *rs16212040* was associated with both gene actions while *rs318126353* was associated only with dominant gene action. Two more SNPs i.e. *rs313298834 (*GGA12) and *rs314011910 (*GGA22) were detected as additive/dominant or dominant markers, respectively. Estimates of *a*, *d* and *|d/a|* for the four SNPs (*rs16212040, rs318126353*, *rs313298834* and *rs314011910*) are shown in Table 2. In line with a purely dominant model where genotypic values are solely determined by the presence or absence of the dominant allele, genotypic means of the minor homozygous and minor heterozygous were found to significantly differ from the major homozygous genotypic means (Table 2). Degree of dominance for the four SNPs ranged from 0.42–0.76 (partial dominance, markers: *rs16212040, rs313298834* and *rs318126353)* to 1.1 (complete dominance, marker: *rs314011910)*. Notably, no marker was associated with over-dominance. We furthermore sought to quantify the joint effect of the combined genotype of the two markers (*rs16212040* and *rs318126353*) retained by LASSO on GGA28 by estimating LS means for the combined genotypes (Table 3). This exercise delivered interesting results as highest EN values were attained for AABB (μ = 138.8, *n* = 9) and ABAA (μ = 138.9, *n* = 71) that could not be attributed to additive allelic effects of individual markers. Specifically, the highest EN estimate for AABB is suggestive of additive-by-additive (AABB) interaction (epistasis) while that of ABAA of additive-by-dominance (ABAA) epistasis. However, due to limited number of observations, especially for the AABB combined genotype (*n* = 9), current results should be treated with caution.

**Table 2:** Estimation of genotypic means (μ ± SE) for EN, additive allelic effects (*a*), dominance deviation (*d*) and degree of dominance (|*d/a*|) for the significant additive/dominant markers

Marker	Genotype (coded as)	Sample size	μ ± SE	***a*** ± SE	***d*** ± SE	|***d/a***|
*rs313298834* (add/dom)	AA (0)	1595	129.9^b^ ± 1.0	3.6^*^ ± 0.8	2.6^NS^ ± 2.0	|2.6/3.6| = 0.72
	AB (1)	245	136.0^a^ ± 2.0			
	BB (2)	746	137.1^a^ ± 1.4			
*rs314011910* (dom)	ΒΒ (0)	2167	133.7^b^ ± 1.0	−3.5^*^ ± 1.0	−3.8^NS^ ± 3.2	|3.8/3.5| = 1.1
	ΑΒ (1)	91	126.4^a^ ± 3.2			
	ΑΑ (2)	328	126.7^a^ ± 2.1			
rs16212040 (add/dom)	AA (0)	1695	135.2^b^ ± 1.0	−5.0^*^ ± 1.3	−2.1^NS^ ± 1.7	|2.1/5.0| = 0.42
	AB (1)	758	128.1^a^ ± 1.3			
	BB (2)	133	125.2^a^ ± 2.6			
rs318126353 (dom)	AA (0)	1583	135.5^b^ ± 1.0	−4.1^*^ ± 1.2	−3.1^*^ ± 1.6	|3.1/4.1| = 0.76
	AB (1)	838	128.3^a^ ± 1.2			
	BB (2)	165	127.3^a^ ± 2.4			

^a,b^ means with different superscripts are statistically different (*p* < 0.05)

^*^statistically significant with *p* < 0.05

^NS^ non statistically significant

**Table 3:** Least squares means (μ ± SE) for EN for combined genotype of markers *rs16212040* and *rs318126353* on GGA28. N is the sample size

Combined Genotype	N	μ ± SE
AA/AA	1512	135.3 ± 1.0
AA/AB	174	134.3 + 2.3
AA/BB	9	138.8 ± 9.6
AB/AA	71	138.9 ± 3.5
AB/AB	639	126.7 ± 1.3
AB/BB	48	129.9 ± 4.2
BB/AB	25	125.7 ± 5.8
BB/BB	108	125.0 ± 2.9

### Positional candidate genes

**Fig. 3:**
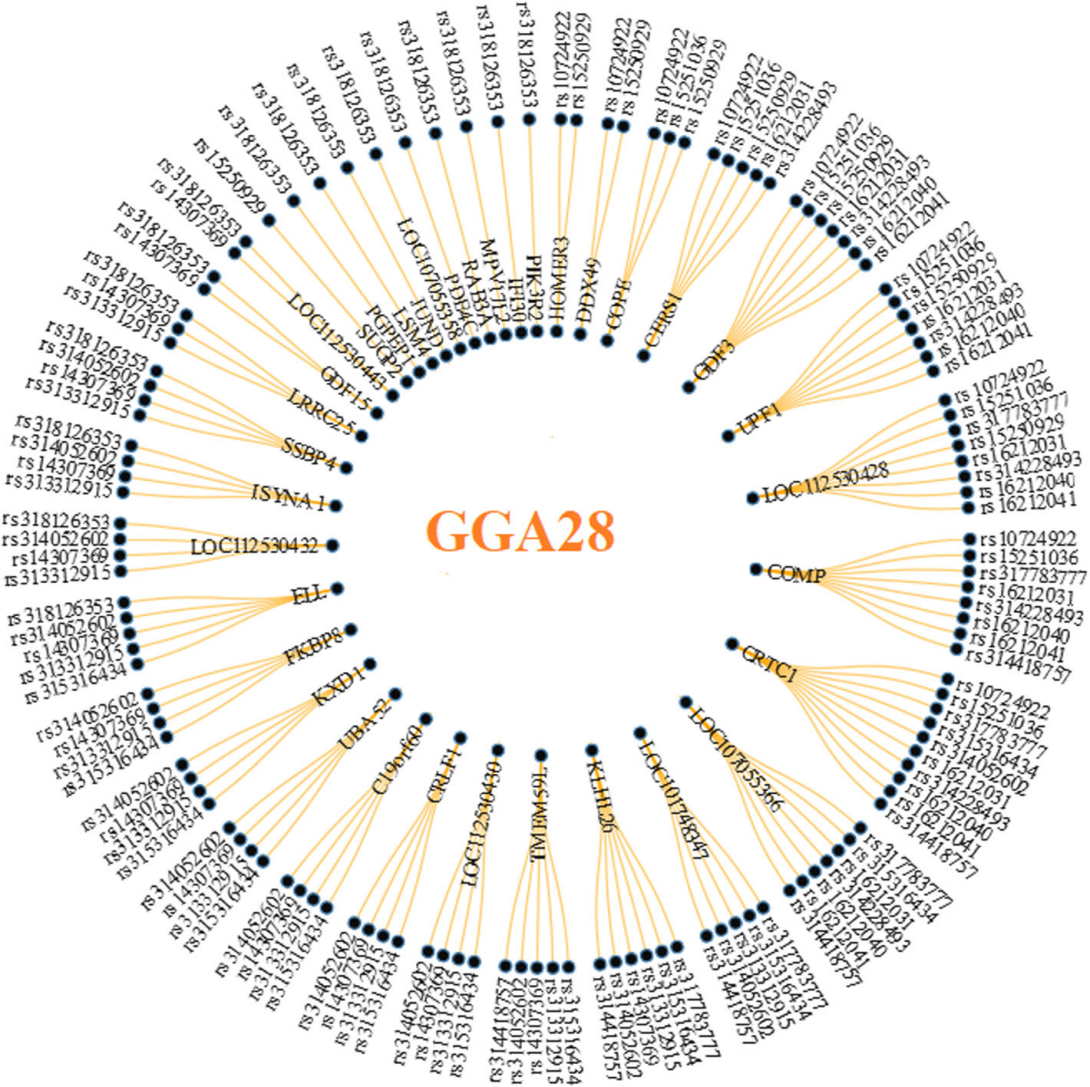
Radial network of significant SNPs associated with positional candidate genes on GGA28. Figure was constructed using the data.tree and networkD3 packages in R (http://www.r-project.org/)

A total number of 57 positional candidate genes (i.e. 43 annotated and 14 LOC genes) were identified within the searched genomic regions (Supplementary Table 1, Additional file 2). The maximum number of genes (*n* = 16) were detected around dominant *rs318126353* (GGA28) while the minimum number of genes (*n* = 6) were identified around 5 SNPs (*rs317783777*, *rs314011910*, *rs16212040, rs16212041* and *rs314418757*). Four additive SNPs (*rs313045367, rs10724922, rs317783777* and *rs315316434*) were located within genes *ARL8A* (GGA26), *UPF1* (GGA28*), CRTC1* (GGA28) and *TMEM59L* (GGA28)*,* while 2 more markers (*rs313312915* and *rs14307369*) resided in gene *ELL* (GGA28). Three dominant SNPs (*rs15250929, rs314052602* and *rs318126353*) lied within genes *DDX49, KXD1 PGPEP1 (*GGA28)*.* Of additive/dominant SNPs, 3 co-localized markers (*rs314228493, rs16212040* and *rs16212041*) were detected within *COMP* (GGA28) and one more (*rs314418757*) within *CRTC1* (GGA28). As 14 significant markers resided in nearby locations on GGA28, 26 out of the 36 positional candidate genes were associated with more than one marker (Fig. 3). The maximum number of SNPs (*n* = 10) were associated with gene *CRTC1*.

### Functional enrichment analysis

A total number of 50 out of the 57 positional candidate genes were recognized by the DAVID tool and used for functional enrichment analysis. The latter analysis revealed the ‘entrainment of circadian clock by photoperiod’ (GO:0043153) as the only significantly (*p* = 0.028) enriched BP with two participating genes (*CRTC1* and *BHLHE40)* (results not shown).

### Prioritized genes

Results of PA are displayed on Table 4. A total number of 10 out of the 43 positional candidate genes were prioritized (overall *p*-value< 0.05) according to the semantic annotations imposed. The majority (*n* = 7) of the prioritized genes resided on GGA28, followed by two genes (*BHLHE40* and *ITPR1*) on GGA12 and one (*ELF3*) on GGA26. On GGA28, the first ranked gene was *GDF15*, followed by *JUND, GDF3, COMP, ELL, CRLF1* and *IFI30*. Notably, two highly ranked genes i.e. *GDF15* (1st) and *GDF3* (4th) belong to the transforming growth factor beta (TGF-β) superfamily. The two genes (*BHLHE40* and *CRTC1)* that participated in GO:0043153 ‘entrainment of circadian clock by photoperiod’ were also prioritized and ranked 2nd and 13th, respectively.

**Table 4:** List of prioritized genes

Rank	***Gene ID***	***Description***	GGA	Overall
				***p***-value
1	*GDF15*	*growth differentiation factor 15*	28	0.019
2	*BHLHE40*	*basic helix-loop-helix family member e40*	12	0.027
3	*JUND*	*JunD proto-oncogene, AP-1 transcription factor subunit*	28	0.029
4	*GDF3*	*growth differentiation factor 3*	28	0.030
5	*COMP*	*cartilage oligomeric matrix protein*	28	0.037
6	*ITPR1*	*inositol 1,4,5-trisphosphate receptor type 1*	12	0.039
7	*ELF3*	*E74 like ETS transcription factor 3*	26	0.040
8	*ELL*	*elongation factor for RNA polymerase II*	28	0.044
9	*CRLF1*	*cytokine receptor like factor 1*	28	0.047
10	*IFI30*	*IFI30, lysosomal thiol reductase*	28	0.048
11	*ISYNA1*	*inositol-3-phosphate synthase 1*	28	0.050
12	*RAB3A*	*RAB3A, member RAS oncogene family*	28	0.051
13	*CRTC1*	*CREB regulated transcription coactivator 1*	28	0.057
14	*GPR37L1*	*G protein-coupled receptor 37 like 1*	26	0.057
15	*PIK3R2*	*phosphoinositide-3-kinase regulatory subunit 2*	28	0.060
16	*GFRA2*	*GDNF family receptor alpha 2*	22	0.061
17	*EDEM1*	*ER degradation enhancing alpha-mannosidase like protein 1*	12	0.069
18	*FKBP8*	*FK506 binding protein 8*	28	0.069
19	*PDE4C*	*phosphodiesterase 4C*	28	0.083
20	*LGR6*	*leucine rich repeat containing G protein-coupled receptor 6*	26	0.086
21	*HOMER3*	*homer scaffolding protein 3*	28	0.112
22	*LSM4*	*LSM4 homolog, U6 small nuclear RNA and mRNA degradation associated*	28	0.114
23	*COPE*	*coatomer protein complex subunit epsilon*	28	0.149
24	*ARL8B*	*ADP ribosylation factor like GTPase 8B*	12	0.150
25	*PTPN7*	*protein tyrosine phosphatase, non-receptor type 7*	26	0.152
26	*PGPEP1*	*pyroglutamyl-peptidase I*	28	0.156
27	*C19orf60 (also known as REX1BD)*	*chromosome 19 open reading frame 60*	28	0.172
28	*SSBP4*	*single stranded DNA binding protein 4*	28	0.192
29	*UBA52*	*ubiquitin A-52 residue ribosomal protein fusion product 1*	28	0.192
30	*UPF1*	*UPF1, RNA helicase and ATPase*	28	0.192
31	*CERS1*	*ceramide synthase 1*	28	0.198
32	*MPV17L2*	*MPV17 mitochondrial inner membrane protein like 2*	28	0.228
33	*DOK2*	*docking protein 2*	22	0.262
34	*XPO7*	*exportin 7*	22	0.292
35	*ARL8A*	*ADP ribosylation factor like GTPase 8A*	26	0.340
36	*DDX49*	*DEAD-box helicase 49*	28	0.345
37	*SUGP2*	*SURP and G-patch domain containing 2*	28	0.345
38	*KLHL26*	*kelch like family member 26*	28	0.345
39	*KIF21B*	*kinesin family member 21B*	26	0.351
40	*KXD1*	*KxDL motif containing 1*	28	0.351
41	*LRRC25*	*leucine rich repeat containing 25*	28	0.588
42	*TMEM59L*	*transmembrane protein 59 like*	28	0.588
43	*PTPRVP*	*protein tyrosine phosphatase, receptor type, V, pseudogene*	26	1.000

## Discussion

### Mode of gene action

This is the first GWAS enlisting a significant number of animals (n ~ 2600) and reporting on genetic variants implicated in the genetic control of EN in broiler breeders. Present results have demonstrated the need to thoroughly exploring the applicability of all possible genetic models when conducting a GWAS. This is particularly important when analyzing quantitative traits such as EN where not only additive but also non-additive e.g. dominant gene action of the causative loci may be fairly anticipated [3]. In line with this expectation, 4 of the 17 significant variants were dominant while 6 more were additive and dominant associations. The latter seems to be a controversial finding, but it can be fairly explained by examining the genotypic means across the examined variants of Table 2. A ‘complete dominant’ genetic model is when |*d*| = |*a*| meaning equal genotypic values for the minor homozygous (μ_ΑΑ_) and the minor heterozygous genοtypes (μ_ΑΒ_) that both differ from the major homozygous genotypic mean (μ_ΒΒ_). This was exactly the case for marker *rs314011910* that was detected only as dominant variant. But what happens in the case of partial dominance (0 < |*d*| < |*a*|)? In such cases (see markers *rs313298834* and *rs16212040* in Table 2) all genotypic means differ (μ_ΑΑ_ ≠ μ_ΑΒ,_ μ_ΑΑ_ ≠ μ_BΒ_ and μ_ΑB_ ≠ μ_BΒ_) meaning that apart from the dominant model, a linear relationship between the genotypic mean values and the number of copies of the minor allele i.e. the additive genetic model may also be applicable. For an excellent interpretation of how least squares regression performs in GWAS in additive and dominant models we refer to Huang and Mackay [12]. So far, we have discussed the applicability of the additive and dominant model, but we have neglected the case of over-dominance (|*d*| > |*a*|). In the latter case, μ_ΑB_ > μ_ΑA_ and μ_ΑB_ > μ_BB_ implying the need of using a different model parameterization by coding the heterozygous genotypes as 1 and the two homozygous genotypes as 0. Due to model parameterization difficulties we could not explore the validity of an over-dominant genetic model here and this may be the reason why no marker has been associated with over-dominance in the current study.

While estimates of genetic effects (additive and/or dominant) are expected unbiased for a few, independent variants, this may not be the case for multiple, highly correlated variants residing on the same haplotype block(s) since the effect(s) may be ‘shared’ by many SNPs. For this reason, it is important to have a parsimonious model involving limited number of regressors (SNPs). To this end, application of the LASSO technique has proved particularly helpful as it has selected only two markers, each one residing in the two LD blocks on GGA28. Then, the next step was to explore whether the two variants interact and, if yes, to portray the exact type of interaction. This exploration has delivered interesting results since non-additive genetic interaction(s) between the two variants could also be detected. Although these findings are based on limited number of observations, they are indicative of potential importance of epistasis in the inheritance of the trait.

### Functional candidate genes

Another intriguing problem that needed to be addressed in the present study was as how to narrow down the list with the 43 positional candidate genes. This post-GWAS step presents an important problem, because the experimental validation of the true causal genetic variants requires considerable costs, effort and time. To address this issue, we performed in silico prioritization analysis (PA) using explicitly two semantic annotations: GO: BP and co-expression. This approach was based on the assumption that co-expressed genes tend to be involved in the same biological process and that expression of functionally related genes should vary concordantly across the various tissues. Typically, gene co-expression networks do not provide information about causality, but they can serve as first proof of their involvement in a particular biological process [13] and can be effectively used for the identification of regulatory genes underlying phenotypes [14]. Following this approach, 10 prioritized genes (*GDF15, BHLHE40, JUND, GDF3, COMP, ITPR1, ELF3, ELL, CRLF1* and *IFI30)* with interesting biological properties were highlighted*.* Genes *GDF15 (growth differentiation factor 15,* placed 1st) and *GDF3 (growth differentiation factor 3, placed 4th*) serve as good examples here since they both belong to the TGF-*β* superfamily genes. In rodents and humans, many factors belonging to the TGF-β superfamily are expressed by ovarian somatic cells and oocytes in a developmental manner and function as intraovarian regulators of folliculogenesis [15]. In humans, *GDF15* is involved in placentation [16], while *GDF3* might affect folliculogenesis by inhibiting the bone morphogenetic protein cytokines [17]. In chickens, *GDF3 (*also known as cVg1) is expressed at the early blastoderm stages of embryonic development [18] while another TGF-β member i.e. *GDF9* is expressed in the ovary and functions on hen granulosa cell proliferation as in mammals [19]. Expression of *BHLHE40 (basic helix-loop-helix family member e40)* in the mouse ovary leads to a circadian gating of cellular processes in the ovary as well as in the hypothalamus during ovulation [20]. *JUND (JunD proto-oncogene, AP-1 transcription factor subunit*) is important for maturation of human ovarian cells [21]. *COMP (cartilage oligomeric matrix protein)* is involved in ovarian follicle development in mice [22] while mutations of *COMP* gene affect chondrogenesis in chickens [23]. *ITPR1 (inositol 1,4,5-trisphosphate receptor type 1)* is involved in the Ca^2+^ transport for supplying eggshell mineral precursors in chicken uterus [24, 25] while *ELF3 (E74 like ETS transcription factor 3)* has been related to the development of chicken oviducts [26] and *ELL (elongation factor for RNA polymerase II)* has been associated with yolk weight [27] in chickens. Notably, the final two nominated candidates i.e. *CRLF1 (cytokine receptor like factor 1)* and *IFI30 (IFI30, lysosomal thiol reductase)* had no documented involvement in reproduction. Such a finding underscores the limitations of in silico PA. In almost every guilt-by-association (GBA)-based prioritization tool, functional annotations of genes refer mainly to human and mouse PPINs (protein-protein interaction networks) [28] neglecting relevant information on livestock species [29] such as that examined here. One more limitation of GBA-based networks relates to their degraded predictive performance for genes with unknown or multiple functions [28].

Of particular interest in this study were genes *BHLHE40* and *CRTC1 (CREB regulated transcription coactivator 1).* Both genes were enriched in the BP of ‘entrainment of circadian clock by photoperiod’ raising the intriguing question as what might be the exact mechanism of their implication in egg production. To answer the question, first we have to provide a short description of the molecular mechanism underlying circadian rhythms (CR). CR are controlled by a pacemaker located within the suprachiasmatic nucleus of the hypothalamus that is entrained to the external light–dark cycle via light input from the retina conveyed via the retinohypothalamic tract [30]. In hens, as in many avian species, exposure to photoperiods of longer than 11.5 h/day causes development and growth of testes and ovarian follicles via rapid induction of the hypothalamo-hypophysial-gonad axis [31]. At the intracellular level, four clock-gene families are reported to be involved in a transcription–translation feedback loop that generates the CR. Gene products of *Clock* and *Bmal1* act as positive components, whereas those of the *Per* and *Cry* genes act as negative ones [32]. With regard to our candidate genes, *BHLHE40 (also known as BHLHB2)* acts as a suppressor of *Clock* and *Bmal1* genes [33] while an entrainment stimulus causes *CRTC1* to induce expression of *Per1* and *Sik1* [34]. As the molecular bases for circadian clocks are highly conserved across species, it is very likely that the avian molecular mechanisms are similar to those expressed in mammals, including humans [31].

In total, 7 (*GDF15, BHLHE40, JUND, GDF3, COMP, ELF3* and *CRTC1*) of the prioritized genes were associated with reproductive traits while 2 (*ITPR1* and *ELL*) were related to egg quality traits. From the above, only 3 genes i.e. *COMP, ELL* and *CRTC1* included significant SNPs. We finally, compared our candidate genes list (Supplementary Table 1, Additional file 2) to a compiled gene list including 271 genes (Supplementary Table 2, Additional file 3) identified in previous GWASs for chicken egg and reproductive traits. This comparison highlighted two common genes i.e. *ELL* and *ARL8A.* Note that the first is among the prioritized candidates (ranked 8th) while the second *ARL8A (ADP ribosylation factor like GTPase 8A)* has been associated with eggshell thickness and eggshell formation [5] in chickens.

## Conclusions

Current results have shown that apart from the additive also the dominant genetic model was of importance for EN in broilers. These results underline the need to thoroughly exploring the applicability of all possible genetic models when performing GWAS for a trait such as that examined here. Detailed follow-up studies are warranted to verify whether the identified genomic markers and the associated candidate genes present true causal genetic entities impacting on the trait. Such studies would entail targeted re-sequencing and molecular characterization of the candidate variants to facilitate the identification of true causal variants.

## Methods

### Data

Genotypic and phenotypic records for 2992 female broiler breeders from a purebred line were provided by Aviagen Ltd. EN records (28 to 50 weeks of age) ranged from 26 to 196 eggs per hen with an average of 132.4 (SD = 29.8). The 600 k Affymetrix® Axiom® high density genotyping array [35] was employed for animal genotyping with a total number of 544,927 SNPs available, dispersed in 29 autosomes (GGA1–28 and GGA33). Quality control (QC) assessment was performed at both sample and marker level. At a sample level, 406 animals were excluded when they had a call rate lower than 0.99 and an autosomal heterozygosity outside the 1.5 inter-quartile range (IQR: 0.013). At a marker level, a number of 305,660 SNPs were removed, based on: call rate (lower than 0.95), minor allele frequency MAF (lower than 0.05) and linkage disequilibrium (LD) pruning (r^2^ values greater than 0.99 within windows of 1 Mb inter-marker distances). Finally, a total of 2586 samples and 239,267 SNPs across 28 autosomes (GGA1–28) were retained for further analyses. QC was performed using the SNP & Variation Suite software (version 8.8.3).

### Marker-trait association analysis

Stepwise regression with forward inclusion and backward elimination of multiple markers (SNPs) [36] was applied to identify SNPs associated with the trait, assuming first an additive and second a dominant gene action for the SNP effects.

Specifically, the following mixed model was used for EN data:

$$ \mathrm{y}=\mathrm{X}\boldsymbol{\upbeta } +\mathbf{w}\boldsymbol{\upalpha } +\mathrm{Z}\mathbf{u}+\mathbf{e} $$

where *y* is the n × 1 vector of phenotypic values of EN for n female broilers, *X* is the n × 53 matrix of fixed effects: hatch (36 classes) and mating group (17 classes), ***β*** is the 53 × 1 vector of corresponding coefficients of fixed effects, ***a*** is the fixed effect for the minor allele of the candidate SNP to be tested for association, ***w*** is the incidence vector relating observations to SNP effects with elements coded as 0 for the major homozygous genotype, 1 for the heterozygote genotype and 2 for the minor homozygous genotype (additive genetic model) and 0 for the major homozygous genotype and 1 for the heterozygous and minor homozygous genotypes (dominant genetic model). Z is the incidence matrix relating observations to the polygenic random effects, ***u*** is the vector of polygenic random effects and ***e*** is the vector of random residuals.

The random effects were assumed to be normally distributed with zero means and the following (co)variance structure:

$$ Var\left[\begin{array}{c}u\\ {}e\end{array}\right]=\left[\begin{array}{cc}G{\sigma}_u^2& 0\\ {}0& I{\sigma}_e^2\end{array}\right] $$

where ${\sigma}_u^2$ and ${\sigma}_e^2$ are the polygenic and error variance components, I is the n x n identity matrix, and G is the n x n genomic relationship matrix (GRM [37]) with elements of pairwise relationship coefficient using the 239,267 SNPs. Τhe genomic relationship coefficient between two individuals j and k, was estimated as follows:

$$ \frac{1}{239,267}\sum \limits_{i=1}^{239,267}\frac{\left({x}_{ij}-2{p}_i\right)\left({x}_{ik}-2{p}_i\right)}{2{p}_i\left(1-2{p}_i\right)} $$

where x_ij_ and x_ik_ represent the number (0, 1, 2 in the additive model and 0, 1, 1 in the dominant model) of the minor allele of the i_th_ SNP of the j_th_ and k_th_ individuals, and p_i_ is the frequency of the minor allele [37].

Statistically significant SNPs per genetic model were selected at the optimal step of the stepwise regression according to the extended Bayesian Information Criterion (eBIC [38]). SNP *p*-values were subsequently corrected for multiple comparisons using the Bonferroni correction method. A SNP was considered as significant at the genome-wide level when its *p*-value was lower than the threshold value 2.09E-07 (0.05/239,267) while a chromosome-wide significant SNP had a *p*-value lower than 0.05/N, where N is the number of SNPs on a given chromosome. All analyses were performed using the SNP & Variation Suite software (version 8.8.3). SNP positions were based on *GRCg6a* assembly (https://www.ncbi.nlm.nih.gov/assembly/GCF_000002315.6 [39], https://www.ncbi.nlm.nih.gov/genome/annotation_euk/Gallus_gallus/104/ [40]).

### Quantile-quantile plots and genomic inflation factors

To characterize the extent to which the observed distribution of the test statistic followed the expected (null) distribution, quantile-quantile (Q-Q) plots were used. These plots in combination with estimates of the genomic inflation factor (*λ*) were used to assess potential systematic bias arising from population structure or the analytical approach [41]. Estimation of *λ* was performed using the SNP & Variation Suite (version 8.8.3).

### Estimation of genomic heritability and proportion of variance explained

Estimation of the genomic heritability was implemented via the realized GRM of 2586 animals derived from the 239,267 SNPs.

The proportion of variance explained by a SNP *k* (*PVE*_*k*_) was also calculated as follows:

$$ {PVE}_k=\frac{mrss_{h0}-{mrss}_k}{mrss_{h0}} $$

where *mrss*_*h*0_ is the Mahalonobis root sum of squares (mrss) for the null hypothesis and *mrss*_*k*_ is the same for marker *k*. All above estimations were performed using the SNP & Variation Suite software (version 8.8.3).

### Identification of significant SNPs under multicollinearity conditions

When multiple markers were present in a specific genomic region, a variable selection method i.e. the Least Absolute Shrinkage and Selection Operator (LASSO) [42] as implemented in procedure GLMSELECT in SAS 9.3 (2012) was applied to identify the most representative markers in the area.

### Estimation of the degree of dominance

Significant SNPs associated with dominant or dominant and additive gene action(s) were further analysed toward estimation of additive allelic effects, dominance deviation and degree of dominance. This analysis was based on estimates of genotype least squares (LS) means by application of a mixed model to the EN data fitting hatch, mating group and the marker as fixed effects and the animal as a random effect. Degrees of freedom were estimated using the Satterthwaite method while the Tukey-Kramer method was used to adjust the *p*-values because of multiple means comparisons. Results of the mixed model analysis are presented as LS means (*μ*) with standard errors (SE). Additive allelic effect (*a*) was defined as half the difference between LS means of the two homozygous genotypes, using the minor homozygous genotypes as reference. Dominance deviation (*d*) was the heterozygous genotype LS mean minus the average of the two homozygous genotype LS means. Finally, degree of dominance was determined as |*d/a|*, where additive = 0–0.20, partial dominance = 0.21–0.80, complete dominance = 0.81–1.20 and over-dominance> 1.20 [43, 44]. This analysis was performed by the MIXED procedure in SAS 9.3 (2012).

### Detection, functional characterization and prioritization of positional candidate genes

We searched within 50 kb downstream and upstream flanking regions of each significant marker for positional candidate genes using the NCBI database (https://www.ncbi.nlm.nih.gov/gene/ [45]) and the *GRCg6a* assembly (https://www.ncbi.nlm.nih.gov/assembly/GCF_000002315.6 [39], https://www.ncbi.nlm.nih.gov/genome/annotation_euk/Gallus_gallus/104/ [40]). Subsequently, the total number of positional candidate genes was subjected to the following analyses: Gene Ontology (GO) Biological Process (BP) enrichment analysis and gene prioritization analysis (PA).

GO enrichment analysis for BP was carried out using the DAVID functional annotation tool (https://david.ncifcrf.gov/, version 6.8) [46] and the *Gallus gallus* species for the input gene list and as genome background. During enrichment analysis, the following settings were used: EASE score (modified Fisher’s exact *p*-value [47]) cutoff = 0.05 and minimum number of genes per GO BP term = 2. GO BPs with *p*-values lower than 0.05 were considered as significantly enriched.

Gene prioritization analysis (PA) of the positional candidate genes followed, using the ToppGene portal (https://toppgene.cchmc.org/prioritization.jsp [48]). PA was based on the functional similarity of the positional candidate genes (test genes) to a training gene list including a total number of 31 genes (Supplementary Table 3, Additional file 4). The latter genes were retrieved from the NCBI database (https://www.ncbi.nlm.nih.gov/gene/ [45]) using the search terms ‘reproduction’ and ‘egg production’ in *Gallus gallus*. Candidate gene prioritization is based on fuzzy functional similarity measures between any two genes and specific semantic annotations imposed. In our study two semantic annotations: ‘GO: Biological Process’ and ‘Coexpression’ were used. A *p*-value for each annotation of a test gene was derived by random sampling of 5000 genes from the whole genome. The partial *p*-values were finally combined into an overall *p*-value using the probability density function. For gene prioritization, there were 30 training genes *(ZNF764L* was omitted) and 43 test genes (positional candidate genes). Not all of the 57 positional candidate genes were included in the analysis because the human homologs could not be found for all of them, especially for LOC genes (*n* = 14)*.* Genes with an overall *p*-value lower than 0.05 were considered as prioritized.

## Supplementary information

**Additional file 1: Supplementary Fig. 1.** Quantile- quantile (Q-Q) plots of the additive (top) and dominant (bottom) SNP effects for EN. Blue dots denote the −log_10_(*p*-value) obtained from the additive (λ = 0.95) and dominant (λ = 0.97) genetic models and the red lines represent the expected values for the null hypothesis under no association. Q-Q plots were constructed with the qqman package [49] in R (http://www.r-project.org/).

**Additional file 2: Supplementary Table 1.** Positional candidate genes for EN.

**Additional file 3: Supplementary Table 2.** Genes obtained by previous GWASs for egg and reproductive traits in *Gallus gallus*.

**Additional file 4: Supplementary Table 3.** List of training genes retrieved by the NCBI database for the ‘reproduction’ and ‘egg production’ queried terms in *Gallus gallus.*

## Data Availability

The data that support the findings of this study are available from Aviagen Ltd. but restrictions apply to the availability of these data, which were used under license for the current study, and so are not publicly available. Data are however available from the authors upon reasonable request and with permission of Aviagen Ltd. In the present study, positions of SNPs and genes were based on *GRCg6a* (Genome Reference Consortium Chicken Build 6a) assembly (https://www.ncbi.nlm.nih.gov/assembly/GCF_000002315.6 [39]) and NCBI *Gallus gallus* Annotation Release 104 (https://www.ncbi.nlm.nih.gov/genome/annotation_euk/Gallus_gallus/104/ [40]). Additionally, the list of positional candidate genes for EN are provided in Supplementary Table 1 (Additional file 2). Furthermore, we provide a list of genes by previous GWASs for egg and reproductive traits in *Gallus gallus* (see Supplementary Table 2, Additional file 3). Finally, we provide the list of training genes (see Supplementary Table 3, Additional file 4) obtained from the NCBI database (https://www.ncbi.nlm.nih.gov/gene/ [45]) for the ‘reproduction’ and ‘egg production’ queried terms for *Gallus gallus*.

## Abbreviations

EN: Egg number
SNP: Single nucleotide polymorphism
GWAS: Genome-wide association studies
PA: Prioritization analysis
GBA: Guilt-by-association
PPINs: Protein-protein interaction networks
CR: Circadian rhythms
QC: Quality control
IQR: Inter-quartile range
MAF: Minor allele frequency
GRM: Genomic relationship matrix
eBIC: Extended Bayesian Information Criterion
LASSO: Least Absolute Shrinkage and Selection Operator
GO: Gene Ontology
BP: Biological process

## References

[1] Hiemstra SJ, Napel J Ten. Study of the impact of genetic selection on the welfare of chickens bred and kept for meat production. Final report of a project commissioned by the European Commission (DG SANCO/2011/12254); 2013.

[2] Abdollahi-Arpanahi R, Pakdel A, Nejati-Javaremi A, Moradi Shahrbabak M, Morota G, Valente BD (2014). Dissection of additive genetic variability for quantitative traits in chickens using SNP markers. J Anim Breed Genet.

[3] Abdollahi-Arpanahi R, Morota G, Valente BD, Kranis A, Rosa GJM, Gianola D (2016). Differential contribution of genomic regions to marked genetic variation and prediction of quantitative traits in broiler chickens. Genet Sel Evol.

[4] Momen M, Mehrgardi AA, Sheikhy A, Esmailizadeh A, Fozi MA, Kranis A (2017). A predictive assessment of genetic correlations between traits in chickens using markers. Genet Sel Evol.

[5] Liao R, Zhang X, Chen Q, Wang Z, Wang Q, Yang C (2016). Genome-wide association study reveals novel variants for growth and egg traits in Dongxiang blue-shelled and white Leghorn chickens. Anim Genet.

[6] Yuan J, Sun C, Dou T, Yi G, Qu L, Qu L (2015). Identification of promising mutants associated with egg production traits revealed by genome-wide association study. PLoS One.

[7] Liu W, Li D, Liu J, Chen S, Qu L, Zheng J (2011). A genome-wide SNP scan reveals novel loci for egg production and quality traits in white Leghorn and Brown-egg dwarf layers. PLoS One.

[8] Liu Z, Sun C, Yan Y, Li G, Wu G, Liu A (2018). Genome-wide association analysis of age-dependent egg weights in chickens. Front Genet.

[9] Sun C, Qu L, Yi G, Yuan J, Duan Z, Shen M (2015). Genome-wide association study revealed a promising region and candidate genes for eggshell quality in an F2 resource population. BMC Genomics.

[10] Yi G, Shen M, Yuan J, Sun C, Duan Z, Qu L (2015). Genome-wide association study dissects genetic architecture underlying longitudinal egg weights in chickens. BMC Genomics.

[11] Shin J-H, Blay S, Graham J, McNeney B (2006). LDheatmap: An *R* Function for Graphical Display of Pairwise Linkage Disequilibria Between Single Nucleotide Polymorphisms. J Stat Softw.

[12] Huang W, Mackay TFC (2016). The genetic architecture of quantitative traits cannot be inferred from variance component analysis. PLoS Genet.

[13] Liseron-Monfils C, Olson A, Ware D. NECorr, a Tool to Rank Gene Importance in Biological Processes using Molecular Networks and Transcriptome Data. bioRxiv. 2018; 10.1101/326868.

[14] van Dam S, Võsa U, van der Graaf A, Franke L, de Magalhães JP (2017). Gene co-expression analysis for functional classification and gene–disease predictions. Brief Bioinform.

[15] Knight PG, Glister C (2006). TGF-β superfamily members and ovarian follicle development. Reproduction.

[16] Fejzo MS, Sazonova OV, Sathirapongsasuti JF, Hallgrímsdóttir IB, Vacic V, MacGibbon KW (2018). Placenta and appetite genes GDF15 and IGFBP7 are associated with hyperemesis gravidarum. Nat Commun.

[17] Shi J, Yoshino O, Osuga Y, Akiyama I, Harada M, Koga K (2012). Growth differentiation factor 3 is induced by bone morphogenetic protein 6 (BMP-6) and BMP-7 and increases luteinizing hormone receptor messenger RNA expression in human granulosa cells. Fertil Steril.

[18] Seleiro EAP, Connolly DJ, Cooke J (1996). Early developmental expression and experimental axis determination by the chicken Vg1 gene. Curr Biol.

[19] Johnson PA, Dickens MJ, Kent TR, Giles JR (2005). Expression and function of growth differentiation Factor-9 in an oviparous species, Gallus domesticus1. Biol Reprod.

[20] Boden MJ, Varcoe TJ, Voultsios A, Kennaway DJ (2010). Reproductive biology of female Bmal1 null mice. Reproduction.

[21] Günthert AR, Gründker C, Hollmann K, Emons G (2002). Luteinizing hormone-releasing hormone induces JunD–DNA binding and extends cell cycle in human ovarian cancer cells. Biochem Biophys Res Commun.

[22] Galdones E, Penalver Bernabe B, Skory RM, Mackovic D, Broadbelt LJ, Shea LD (2011). Ovarian Expression of Cartilage Oligomeric Matrix Protein as a Potential Biomarker of Antral Follicle Development in the Mouse. Biol Reprod.

[23] Roman-Blas J, Dion AS, Seghatoleslami MR, Giunta K, Oca P, Jimenez SA (2010). MED and PSACH COMP mutations affect chondrogenesis in chicken limb bud micromass cultures. J Cell Physiol.

[24] Duan Z, Chen S, Sun C, Shi F, Wu G, Liu A (2015). Polymorphisms in ion transport genes are associated with eggshell mechanical property. PLoS One.

[25] Jonchère V, Brionne A, Gautron J, Nys Y (2012). Identification of uterine ion transporters for mineralisation precursors of the avian eggshell. BMC Physiol.

[26] Song G, Seo HW, Choi JW, Rengaraj D, Kim TM, Lee BR (2011). Discovery of candidate genes and pathways regulating oviduct development in Chickens1. Biol Reprod.

[27] Sun C, Lu J, Yi G, Yuan J, Duan Z, Qu L (2015). Promising loci and genes for yolk and ovary weight in chickens revealed by a genome-wide association study. PLoS One.

[28] Gillis J, Pavlidis P (2012). “Guilt by association” is the exception rather than the rule in gene networks. PLoS Comput Biol.

[29] Kominakis A, Hager-Theodorides AL, Zoidis E, Saridaki A, Antonakos G, Tsiamis G (2017). Combined GWAS and ‘guilt by association’-based prioritization analysis identifies functional candidate genes for body size in sheep. Genet Sel Evol.

[30] Hughes S, Jagannath A, Hankins MW, Foster RG, Peirson SN (2015). Photic regulation of clock systems. Methods Enzymol.

[31] Cassone VM (2014). Avian circadian organization: a chorus of clocks. Front Neuroendocrinol.

[32] Honma S, Kawamoto T, Takagi Y, Fujimoto K, Sato F, Noshiro M (2002). *Dec1* and *Dec2* are regulators of the mammalian molecular clock. Nature.

[33] Nakashima A, Kawamoto T, Honda KK, Ueshima T, Noshiro M, Iwata T (2008). DEC1 modulates the circadian phase of clock gene expression. Mol Cell Biol.

[34] Jagannath A, Butler R, Godinho SIH, Couch Y, Brown LA, Vasudevan SR (2013). The CRTC1-SIK1 pathway regulates entrainment of the circadian clock. Cell.

[35] Kranis A, Gheyas AA, Boschiero C, Turner F, Yu L, Smith S (2013). Development of a high density 600K SNP genotyping array for chicken. BMC Genomics.

[36] Segura V, Vilhjálmsson BJ, Platt A, Korte A, Seren Ü, Long Q (2012). An efficient multi-locus mixed-model approach for genome-wide association studies in structured populations. Nat Genet.

[37] VanRaden PM (2008). Efficient methods to compute genomic predictions. J Dairy Sci.

[38] Chen J, Chen Z (2008). Extended Bayesian information criteria for model selection with large model spaces. Biometrika.

[39] GRCg6a in NCBI database. https://www.ncbi.nlm.nih.gov/assembly/GCF_000002315.6. Accessed 21 Apr 2019.

[40] NCBI *Gallus gallus* Annotation Release 104. https://www.ncbi.nlm.nih.gov/genome/annotation_euk/Gallus_gallus/104/. Accessed 21 Apr 2019.

[41] Yang J, Weedon MN, Purcell S, Lettre G, Estrada K, Willer CJ (2011). Genomic inflation factors under polygenic inheritance. Eur J Hum Genet.

[42] Zou H (2006). The adaptive Lasso and its Oracle properties. J Am Stat Assoc.

[43] Babu R, Nair SK, Kumar A, Rao HS, Verma P, Gahalain A (2006). Mapping QTLs for popping ability in a popcorn × flint corn cross. Theor Appl Genet.

[44] Mori A, Tsuda Y, Takagi M, Higa Y, Severson DW (2016). Multiple QTL determine dorsal abdominal scale patterns in the mosquito Aedes aegypti. J Hered.

[45] Gene in NCBI database. https://www.ncbi.nlm.nih.gov/gene/. Accessed 21 Apr 2019.

[46] Dennis G, Sherman BT, Hosack DA, Yang J, Gao W, Lane H (2003). DAVID: database for annotation, visualization, and integrated discovery. Genome Biol.

[47] Hosack DA, Dennis G, Sherman BT, Lane HC, Lempicki RA, Lempicki RA (2003). Identifying biological themes within lists of genes with EASE. Genome Biol.

[48] Chen J, Bardes EE, Aronow BJ, Jegga AG (2009). ToppGene Suite for gene list enrichment analysis and candidate gene prioritization. Nucleic Acids Res.

[49] Turner SD. qqman: an R package for visualizing GWAS results using Q-Q and manhattan plots. bioRxiv. 2014; 10.1101/005165.

