# Supplementary figures and images for: Deciphering the mode of action and position of genetic variants impacting on egg number in broiler breeders
Source: BMC Genomics. 2020 Jul 24;21:512. doi: 10.1186/s12864-020-06915-1 (PMC7379350; doi:10.1186/s12864-020-06915-1)

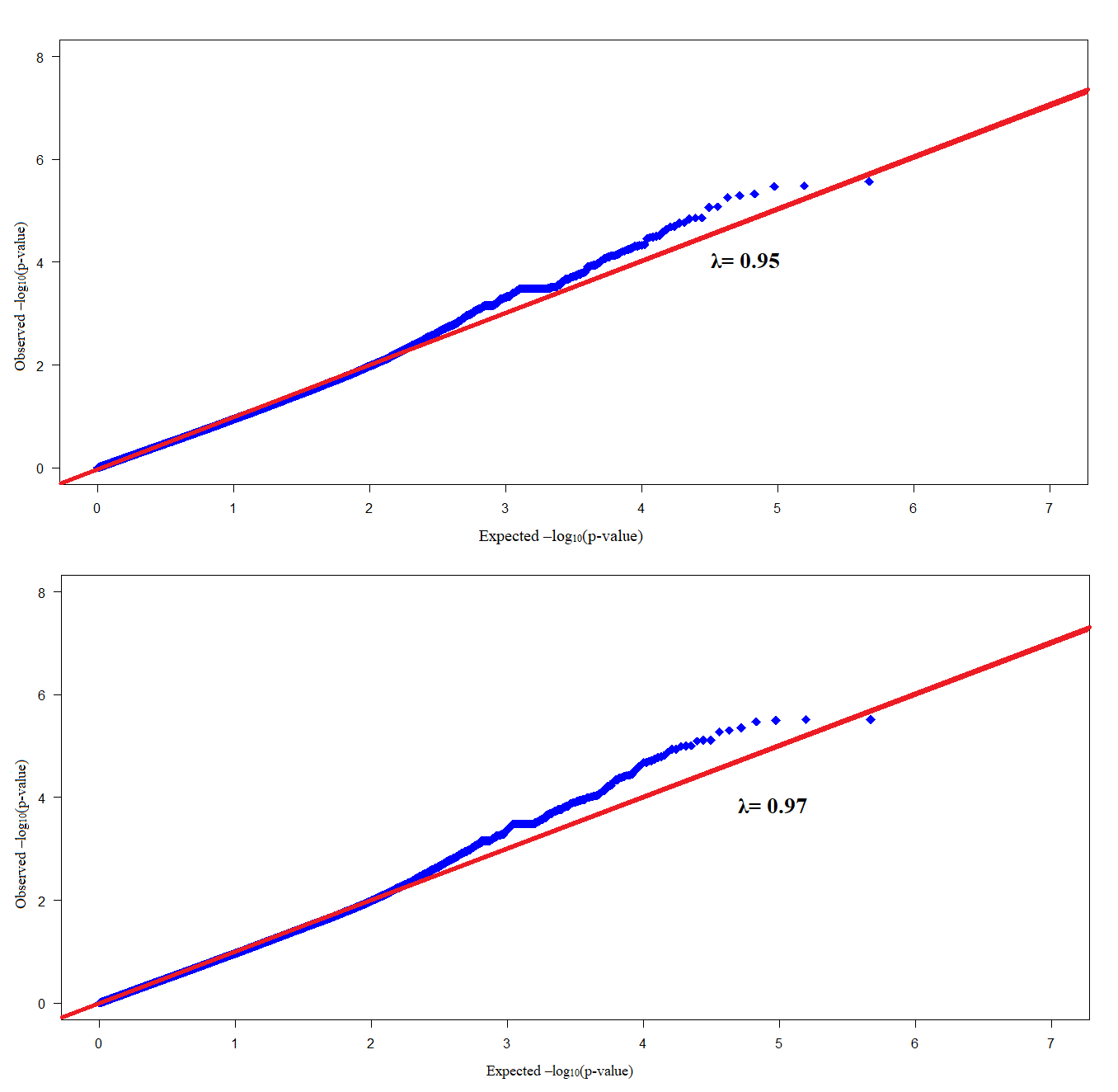

Supplement: Supplementary file 1 — Additional file 1: Supplementary Fig. 1. Quantile- quantile (Q-Q) plots of the additive (top) and dominant (bottom) SNP effects for EN. Blue dots denote the −log10(p-value) obtained from the additive (λ = 0.95) and dominant (λ = 0.97) genetic models and the red lines represent the expected values for the null hypothesis under no association. Q-Q plots were constructed with the qqman package [49] in R (http://www.r-project.org/). [file 12864_2020_6915_MOESM1_ESM.png]
