# Supplementary material for: Deciphering the mode of action and position of genetic variants impacting on egg number in broiler breeders
Source: BMC Genomics. 2020 Jul 24;21:512. doi: 10.1186/s12864-020-06915-1 (PMC7379350; doi:10.1186/s12864-020-06915-1)
Supplement: Supplementary file 3 — Additional file 3: Supplementary Table 2. Genes obtained by previous GWASs for egg and reproductive traits in Gallus gallus. [file 12864_2020_6915_MOESM3_ESM.pdf]

Supplementary Table 2. Genes obtained by previous GWA studies for egg and reproductive traits in *Gallus gallus*

Gene	Trait	Reference
AJAP1<br>*TNFRSF9*<br>*C1ORF174*<br>*CAMTA1*<br>*CEP104*<br>*PDAI1*<br>*SDHB*<br>*DJ-1*<br>*PADI3*<br>*MRPS16*	eggshell blueness	Darwish et al. 2019 [1]
ZNF704<br>*CA2*<br>*RFT1*<br>*PRKCD*<br>*PDGFD*<br>*DYNC2H1*<br>*DCUN1D5*<br>*FGF9*<br>*KIAA1468*<br>*PHLPP1*<br>*ZCCHC2*<br>*TLL1*	yield of extraembryonic fluid, age at first egg, egg weight	Kudinov et al. 2019 [2]
NCAPG<br>*FGFPB1*<br>*KPNA3*<br>*CDC25A*<br>*WDR48*<br>*BST1*<br>*THSD7B*	egg albumen quality	Qu et al. 2019[3]
DLEU7<br>*MIR15A*<br>*CECR2*<br>*MEIS1*<br>*SPRED2*<br>*RNASEH2B*<br>*KCNRG*<br>*SPRYD7*<br>*CECR1*<br>*CECR5*	egg weight	Liu et al. 2018[4]
MSX2<br>*DRD1*	egg quality	Liu et al. 2018[5]

RHOA<br>*SDF4*<br>*TNFRSF4*<br>*TTLL10*<br>*LOC419425*<br>*MIR429*		
FAM184B<br>*HTT*<br>*KCNH7*<br>*CDC42BPA*<br>*KCNIP4*<br>*GJA5*<br>*CBFB*<br>*GPC6*<br>*COG6*<br>*PPARGC1A*<br>*CNKSR2*<br>*MED30*<br>*STK31*	age at first egg , body weight at first egg, egg weight, egg number	Fan et al. 2017[6]
IGFBP3<br>*GORAB*<br>*CCKAR*	oviduct development	Shen et al. 2017[7]
RGS3<br>*AMH*<br>*DCLK1*<br>*NBEA*<br>*SMAD9*	follicle number	Shen et al. 2017[8]
ADIPOR2<br>*LRTM2*<br>*SOX5*<br>*CMAS*<br>*ERC1*<br>*CHUNK-1*<br>*STRAP*<br>*PLCZ1*<br>*AEBP2*<br>*SLCO1A2*<br>*IAPP*<br>*KCNJ8*<br>*GYS2*<br>*SPX*<br>*KRAS*<br>*IQSEC3*<br>*SLC6A12*<br>*WNK1*	eggshell ultrastructure	Duan et al. 2016[9]

CACNA2D4<br>*DCP1B*<br>*CACNA1C*<br>*BCL2L13*<br>*MICAL3*<br>*BPGM*<br>*CALD1*<br>*RERG*<br>*PTPRO*<br>*EPS8*<br>*DERA*<br>*MGST1*<br>*LMO3*<br>*RERGL*<br>*PLEKHA5*<br>*SLCO1C1*<br>*PYROXD1*<br>*BCAT1*<br>*C2CD5*<br>*ST8SIA1*<br>*LDHB*<br>*LRMP*<br>*LYRM5*<br>*RAD52*<br>*WNT5B*<br>*AKR*<br>*CAPZA3*<br>*TMTC1*<br>*TUBAT*<br>*BICD1*<br>*DENND5B*<br>*SLC6A13*<br>*B4GALNT3*<br>*RASSF8*<br>*SLC15A5*<br>*WASH1*<br>*CYB5R3*<br>*ITM2C*<br>*KNDC1*<br>*GPR123(ADGRA1)*		
DGKQ<br>*SHROOM3*<br>*RBPJ*<br>*TRPC6*<br>*C4*	total egg numbers , egg weight, eggshell thickness, eggshell color	Liao et al. 2016[10]

TRPM8<br>*MCCC2*<br>*PTPRD*<br>*AUTS2*<br>*ITGA6*<br>*PLCB1*<br>*CCDC82*<br>*LGSN*<br>*EPHX2*<br>*ANKMY1*<br>*APEM1*<br>*POLA1*<br>*DAPK1*<br>*SMYD3*<br>*ARL8A*<br>*LOC10751519*<br>*LOC100857741*<br>*C5H15ORF29*<br>*LOC101751216*<br>*MEF2A*<br>*ELFN1*<br>*LAMC3*<br>*HAS2*<br>*PRL*<br>*PXDN*<br>*SLC8A1*<br>*KATNBL1*		
DOTL1<br>*GALC*<br>*RPA2*<br>*ACTL9*<br>*ZAP70*<br>*BRCA2*<br>*RFX2*<br>*ONECUT3*<br>*ZAR1*<br>*REXO1*<br>*NRTN*<br>*EML4*<br>*KCNG3*<br>*ADAMTS10*<br>*SCAMP4*<br>*ABHD17A*<br>*ELL*<br>*ENSGALG00000028314*	yolk and ovary weights	Sun et al. 2015[11]

RGS3F<br>*OAZ*<br>*C19orf35*<br>*AP3D1*<br>*MYO1F*<br>*MUC16*<br>*SPG20*<br>*STARD13*<br>*NEK5*<br>*NEK3*<br>*CKAP2*<br>*DHRS12*<br>*ACER1b*<br>*ACSBG2*<br>*ACER1*		
ITPR2<br>*PIK3C2G*<br>*RECQL*<br>*ABCC9*<br>*CASC1*	eggshell quality	Sun et al. 2015[12]
CAB39L<br>*FOXO1*<br>*CDADC1*	egg weight	Yi et al. 2015[13]
GTF2A1<br>*STON2*<br>*CLSPN*<br>*FARSB*<br>*KIAA1549*<br>*CALM1*<br>*Gprotein*<br>*GNB2L1*<br>*TRIM27*<br>*GRIK3*<br>*B-G*<br>*TSN*<br>*THYN1*<br>*SEL1L*<br>*OPCML*<br>*SPPL2B*<br>*TCF3*<br>*TAP2*<br>*PLXNB2*<br>*RPS29*<br>*METAP1D*<br>*TLK1*	egg number , egg laying rate and age at first egg	Yuan et al. 2015[14]

ZAK<br>*PPP1R9B*<br>*TRA2B*<br>*ZC3H12A*<br>*RRAGC*<br>*STMN1*<br>*PNISR*<br>*KIAA0776*<br>*ATG5*<br>*NRXN3*<br>*MOGAT1*<br>*MIR1741*<br>*SMG6*<br>*ADORA2B*<br>*SVOPL*<br>*HIPK2*<br>*TAB1*<br>*PFKP*<br>*PPP4R1L*<br>*C3AR1*<br>*PDE3A*<br>*ARNTL2*<br>*PPFIBP1*<br>*HTR2C*<br>*LOC101749001*<br>*DMC1*<br>*ZC3H14*<br>*KCNK10*<br>*TTC7B*<br>*DIO2*<br>*C5H14ORF159*<br>*TTC8*<br>*LOC423393*<br>*NEURL1B*<br>*CDKAL1*<br>*SOX4*<br>*MIR216B*<br>*MIR7463*<br>*RTN4IP1*<br>*MIR6583*<br>*CCDC88C*<br>*POU3F1*		
GRB14<br>*GALNT1*<br>*ODZ2*	egg production and quality	Liu et al. 2011[15]

ZNF536<br>*ATM*<br>*LOC418918*<br>*ENOX1*<br>*BLK*		

References

1. Darwish HYA, Dalirsefat SB, Dong X, Hua G, Chen J, Zhang Y, et al. Genome-wide association study and a post replication analysis revealed a promising genomic region and candidate genes for chicken eggshell blueness. PLoS One. 2019;14:e0209181. doi:10.1371/journal.pone.0209181.

2. Kudinov AA, Dementieva N V., Mitrofanova O V., Stanishevskaya OI, Fedorova ES, Larkina TA, et al. Genome-wide association studies targeting the yield of extraembryonic fluid and production traits in Russian White chickens. BMC Genomics. 2019;20:270. doi:10.1186/s12864-019-5605-5.

3. Qu L, Shen M, Guo J, Wang X, Dou T, Hu Y, et al. Identification of potential genomic regions and candidate genes for egg albumen quality by a genome-wide association study. Arch Anim Breed. 2019;62:113–23. doi:10.5194/aab-62-113-2019.

4. Liu Z, Sun C, Yan Y, Li G, Wu G, Liu A, et al. Genome-Wide Association Analysis of Age-Dependent Egg Weights in Chickens. Front Genet. 2018;9:128. doi:10.3389/fgene.2018.00128.

5. Liu Z, Sun C, Yan Y, Li G, Shi F, Wu G, et al. Genetic variations for egg quality of chickens at late laying period revealed by genome-wide association study. Sci Rep. 2018;8:10832. doi:10.1038/s41598-018-29162-7.

6. Fan QC, Wu PF, Dai GJ, Zhang GX, Zhang T, Xue Q, et al. Identification of 19 loci for reproductive traits in a local Chinese chicken by genome-wide study. Genet Mol Res. 2017;16. doi:10.4238/gmr16019431.

7. Shen M, Qu L, Ma M, Dou T, Lu J, Guo J, et al. A genome-wide study to identify genes responsible for oviduct development in chickens. PLoS One. 2017;12:e0189955. doi:10.1371/journal.pone.0189955.

8. Shen M, Sun H, Qu L, Ma M, Dou T, Lu J, et al. Genetic Architecture and Candidate Genes Identified for Follicle Number in Chicken. Sci Rep. 2017;7:16412. doi:10.1038/s41598-017-16557-1.

9. Duan Z, Sun C, Shen M, Wang K, Yang N, Zheng J, et al. Genetic architecture dissection by genome-wide association analysis reveals avian eggshell ultrastructure traits. Sci Rep. 2016;6:28836. doi:10.1038/srep28836.

10. Liao R, Zhang X, Chen Q, Wang Z, Wang Q, Yang C, et al. Genome-wide association study reveals novel variants for growth and egg traits in Dongxiang blue-shelled and White Leghorn chickens. Anim Genet. 2016;47:588–96. doi:10.1111/age.12456.

11. Sun C, Lu J, Yi G, Yuan J, Duan Z, Qu L, et al. Promising Loci and Genes for Yolk and Ovary Weight in Chickens Revealed by a Genome-Wide Association Study. PLoS One. 2015;10:e0137145. doi:10.1371/journal.pone.0137145.

12. Sun C, Qu L, Yi G, Yuan J, Duan Z, Shen M, et al. Genome-wide association study revealed a promising region and candidate genes for eggshell quality in an F2 resource population. BMC Genomics. 2015;16:565. doi:10.1186/s12864-015-1795-7.

13. Yi G, Shen M, Yuan J, Sun C, Duan Z, Qu L, et al. Genome-wide association study dissects genetic architecture underlying longitudinal egg weights in chickens. BMC Genomics. 2015;16:746. doi:10.1186/s12864-015-1945-y.

14. Yuan J, Sun C, Dou T, Yi G, Qu L, Qu L, et al. Identification of Promising Mutants Associated with Egg Production Traits Revealed by Genome-Wide Association Study. PLoS One. 2015;10:e0140615. doi:10.1371/journal.pone.0140615.

15. Liu W, Li D, Liu J, Chen S, Qu L, Zheng J, et al. A Genome-Wide SNP Scan Reveals Novel Loci for Egg Production and Quality Traits in White Leghorn and Brown-Egg Dwarf Layers. PLoS One. 2011;6:e28600. doi:10.1371/journal.pone.0028600.
