# Supplementary material for: Deciphering the mode of action and position of genetic variants impacting on egg number in broiler breeders
Source: BMC Genomics. 2020 Jul 24;21:512. doi: 10.1186/s12864-020-06915-1 (PMC7379350; doi:10.1186/s12864-020-06915-1)
Supplement: Supplementary file 4 — Additional file 4: Supplementary Table 3. List of training genes retrieved by the NCBI database for the ‘reproduction’ and ‘egg production’ queried terms in Gallus gallus. [file 12864_2020_6915_MOESM4_ESM.pdf]

Supplementary Table 3. List of training genes retrieved by the NCBI database for the 'reproduction' and 'egg production' queried terms in *Gallus gallus*.

Gene ID	***Description***	**Queried term**
GNRH1	*gonadotropin releasing hormone 1*	reproduction
IGF1	*insulin like growth factor 1*	
MSTN	*myostatin*	
TGFB2	*transforming growth factor beta 2*	
GHRL	*ghrelin, preproghrelin*	
PCK1	*phosphoenolpyruvate carboxykinase 1*	
IGF2	*insulin like growth factor 2*	
LITAF	*lipopolysaccharide induced TNF factor*	
STAT3	*signal transducer and activator of transcription 3*	
TSHR	*thyroid stimulating hormone receptor*	
ACTA2	*actin, alpha 2, smooth muscle, aorta*	
LAMA1	*laminin subunit alpha 1*	
STAT5A	*signal transducer and activator of transcription 5A*	
ZNF764L	*zinc finger protein 764-like*	
PRL	*prolactin*	egg production
GH	*growth hormone*	
PPARG	*peroxisome proliferator-activated receptor gamma*	
PRLR	*prolactin receptor*	
HMGCR	*3-hydroxy-3-methylglutaryl-CoA reductase*	
GDF9	*growth differentiation factor 9*	
POSTN	*periostin*	
NPY	*neuropeptide Y*	
GNRHR	*gonadotropin-releasing hormone receptor*	
FOXL2	*forkhead box L2*	
RARRES1	*retinoic acid receptor responder 1*	
THRSP	*thyroid hormone responsive*	
BMP15	*bone morphogenetic protein 15*	
CETP	*cholesteryl ester transfer protein*	
PDGFRL	*platelet derived growth factor receptor like*	
C9ORF152	*chromosome 2 open reading frame, human C9orf152*	
NCOA1	*nuclear receptor coactivator 1*	
